# Echocardiography Evaluation of Left Ventricular Diastolic Function in Elderly Women with Metabolic Syndrome

**DOI:** 10.1515/med-2019-0073

**Published:** 2019-08-31

**Authors:** Jin-Wook Chung, Dong-il Seo, Yoonjung Park, Wi-Young So

**Affiliations:** 1 College of Humanities and Arts, Sports and Health Care Major, Korea National University of Transportation, 50 Daehak-ro, Chungju-si, Chungbuk 27469, Republic of Korea; 2 Department of Internal Medicine, Division of Cardiology, School of Medicine, Keimyung University, Daegu, Republic of Korea; 3 Department of Sport Science, Dongguk University, Gyeongju, Republic of Korea; 4 Department of Health and Human Performance, College of Liberal Arts and Social Sciences, University of Houston, Texas, USA

**Keywords:** Metabolic syndrome, Physical activity, Ventricular function

## Abstract

To date, we found no published reports on the effects of metabolic syndrome and physical activity levels on left ventricular (LV) diastolic function in elderly women aged over 65 years. Our study involved patients with echocardiographically normal LV ejection fractions (≥50%) and normal LV dilatation diameters (≤55 mm). Elderly women with metabolic syndrome (n = 20) and healthy elderly women (n = 17) were selected and assessed with the National Cholesterol Education Program Adult Treatment Panel III, a metabolic syndrome diagnostic instrument. We compared the LV function indices and physical activity levels according to the presence (metabolic syndrome group) or absence (normal group) of metabolic syndrome. The LV end-systolic (LVES) diameter was significantly smaller (p = 0.037) and LV outflow tract (LVOT) diameter was significantly larger (p = 0.030) in the metabolic syndrome group. The left arterial dimension at end-systole (p = 0.024), left arterial volume (LAV) index (p = 0.015), early peak mitral inflow velocity (E, p = 0.031), early diastolic mitral annulus motion velocity (Eʹ-septal, p = 0.044), (Eʹ-lateral, p = 0.008), and E/late peak mitral inflow velocity ratio (E/A, p = 0.006) values were significantly lower and physical activity levels (p = 0.034) were significantly higher in the metabolic syndrome group. These results indicated that the metabolic syndrome group had relatively high physical activity levels compared to the normal group, which may have positively affected the LVES, LVOT, left atrial volume index, E, Eʹ, and E/A values.

## 1 Introduction

The cause of metabolic syndrome has not been clearly elucidated; however, insulin resistance has been commonly reported as the pathophysiological mechanism [1]. Symptoms of metabolic syndrome include abdominal obesity, abnormal serum glucose levels, hypertension, high triglyceride levels, low high-density lipoprotein (HDL) cholesterol levels, and the presence of cardiovascular risk factors [2, 3]. The prevalence and associated mortality rates of cardiovascular diseases are higher among elderly people, especially women, than among young people [4]. Although the lack of physical activity among elderly people increases their risk of metabolic syndrome and cardiovascular diseases, regular physical activity can help improve their health by reducing hypertension and decreasing low-density lipoprotein cholesterol levels [5].

Obesity results in structural and functional cardiac changes that are caused by the increased amounts of adipose tissue and oxygen demand. These changes result in increased cardiac output and, consequently, increased left ventricular (LV) mass [6]. Higher degrees of obesity, specifically abdominal obesity, are reported to lessen LV relaxation [7]. In addition, if LV systolic dysfunction occurs, a high left atrial pressure is required to maintain the cardiac output, thereby increasing LV diastolic pressure, which eventually promotes heart failure or other cardiovascular diseases [8, 9].

Regular physical activity is an important factor for the prevention and treatment of metabolic syndrome [10] and positively affects cardiovascular risk factors [11]. LV relaxation function can be affected by physical activity habit and is also related to metabolic syndrome; thus, identifying the relationship between physical activity levels and LV relaxation function according to the presence of metabolic syndrome in elderly females can be meaningful basic data to curtail medical expenses. In particular, various studies have reported the benefits of physical activity for improving metabolic syndrome and cardiovascular risk factors in elderly people [12, 13]. However, none have reported the effects of metabolic syndrome and physical activity levels on LV diastolic function in elderly women. Therefore, in this study, we compared physical activity levels among elderly women with and without metabolic syndrome and analyzed the factors related to LV diastolic function in these groups of women.

## 2 Methods

### 2.1 Patients

In this study, we examined 190 patients who visited a health promotion center (Dongguk University, Gyeongju, Republic of Korea) for a health check-up and volunteered for the study. They understood the purpose of the study and provided consent. Thereafter, they underwent carotid intima-media thickness measurements and echocardiography between September and December 2015 at the center. The study included only patients with echocardiographically normal LV ejection fraction (LVEF, ≥50%) and normal LV dilatation diameter (≤55 mm). Patients with cardiovascular disease and complications (angina, myocardial infarction, heart failure, and stroke), valvular disease, and arrhythmia were excluded. Patients with diseases or those who were taking medications for the treatment of diseases that could affect LV diastolic function, such as hypertension, diabetes, hyperlipidemia, anemia, and thyroid or renal dysfunction, were also excluded. Moreover, they did not participate regularly in exercise program, no musculoskeletal diseases, were non-smokers, and did not take any dietary supplements. Patients were assessed using the National Cholesterol Education Program Adult Treatment Panel-III—a metabolic syndrome diagnostic instrument [14].

We compared LV function indices and physical activity levels according to presence or absence of metabolic syndrome in elderly women. The Institutional Review Board of Dongguk University Hospital approved this study.

### 2.2 Measurements

Blood pressure was measured after patients rested for ≥10 minutes; once the readings were stable, pulse rates and systolic and diastolic blood pressures were measured with an automatic sphygmomanometer (Space-Labs Medical, model 90207, USA) attached to the right upper arm. All patients fasted for >8 hours before their blood samples were collected for glucose (blood sugar), triglyceride, and HDL cholesterol level determinations. Body weights and heights were measured by the BSM 330 (Biospace, Seoul, Korea), with each patient in the standing position and wearing an examination gown but without shoes. The body mass index (kg/m^2^) was calculated by dividing the body weight by the squared height value. Waist circumference was measured to the nearest 0.1 cm at a point between the lowest rib and the iliac crest while the patient was in the standing position, in order to assess abdominal obesity. In women, abdominal obesity is indicated by a waist circumference of >85 cm, as defined by the Korean Society for the Study of Obesity [15].

A structured lifestyle questionnaire was administered to patients to determine their smoking and physical activity habits. For smoking, participants were classified as “never smoked,” “past smokers,” and “present smokers.” Physical activity was assessed using the International Physical Activity Questionnaire, with the amount of physical activity scored as metabolic equivalent task-minutes/week [16].

The Vivid 7 Dimension instrument (GE Medical, Chicago, IL, USA) was used for echocardiography. M-mode was measured from the papillary muscles in the LV parasternal short-axis views and was used to measure the left atrial dimension (LAD), LV end-diastolic dimension (LVD diastole), LV end-systolic dimension (LVD systole), LVEF, and left atrial volume (LAV). The LV mass index was determined by dividing the body surface area by the mass value. To calculate the LV systolic function index, the ratio between the early (E) and late (A) peak mitral inflow velocity (E/A ratio) was determined; the early (Eʹ) and late (Aʹ) diastolic mitral annulus motion velocities were measured at the mitral septum, and the deceleration time (DT) was determined.

### 2.3 Statistical analysis

Descriptive variables are presented as means ± standard deviations. Data analyses were performed via independent t-tests, with statistical significance set at p < 0.05. SPSS 21.0 (IBM, Armonk, NY, USA) was used to perform all analyses.

## 3 Results

Among the 90 patients (36 men and 54 women) who met the inclusion criteria, 20 elderly women with metabolic syndrome and 17 healthy elderly women were selected for inclusion in the study. With the exception of patients with two risk factors for metabolic syndrome, normal individuals were selected. General characteristics of patients in each group are shown in Table 1.

**Table 1:** General characteristics of the patients

		Metabolic syndrome (n = 20)	Normal (n = 17)	p
Age (years)		74.6 ± 4.3	71.4 ± 5.3	0.051
Height (cm)		152.0 ± 7.0	153.0 ± 6.0	0.874
Weight (kg)		59.3 ± 10.2	54.8 ± 7.8	0.143
Body mass index (kg/m^2^)		25.6 ± 3.7	23.7 ± 3.4	0.105
Systolic blood pressure (mmHg)		135.8 ± 14.0	126.9 ± 14.5	0.067
Diastolic blood pressure (mmHg)		80.7 ± 11.4	77 ± 10.6	0.324
Waist circumference (cm)		89.0 ± 10.7	81.0 ± 8.5	0.019^*^
Blood glucose level (mg/dL)		120.1 ± 43.3	88.7 ± 7.2	0.006^**^
Triglyceride level (mg/dL)		173.3 ± 64.3	94.5 ± 29.7	<0.001^***^
High density lipoprotein cholesterol level (mg/dL)		56.4 ± 16.5	65.2 ± 12.2	0.077

Data are expressed as means ± standard deviations ^*^p < 0.05, ^**^ *p*< 0.01, ^***^p < 0.001, as tested using the independent *t*-tes

The LV function indices and physical activity levels are shown in Table 2. The mean LV end-systolic diameter (LVES) was significantly smaller in patients in the metabolic syndrome group compared to in those in the normal group (p = 0.037). Furthermore, the mean LV outflow tract (LVOT) diameter was significantly larger in the metabolic syndrome group (p = 0.030). However, the mean left atrial volume (LAV) was significantly lower in the metabolic syndrome group than for the normal group (p = 0.024), as was the LAV index (p = 0.015). Further, the mean E (p = 0.031), Eʹ-septal (p = 0.044), Eʹ-lateral (p = 0.008), and E/A ratio (p = 0.006) values were all significantly lower, and mean physical activity level was significantly higher (p = 0.034) in the metabolic syndrome group.

**Table 2:** Patient left ventricular function indices and physical activity levels

Group	Metabolic syndrome (n = 20)	Normal (n = 17)	*p*
LVEF (%)	63.50 ± 1.90	62.65 ± 2.01	0.203
LVED (mm)	46.50 ± 3.49	46.00 ± 11.54	0.855
LVES (mm)	26.60 ± 2.74	28.71 ± 3.18	0.037^*^
IVSD (mm)	10.55 ± 1.67	10.06 ± 1.85	0.402
IVSS (mm)	16.25 ± 1.97	15.41 ± 1.77	0.185
PWTD (mm)	10.15 ± 1.60	9.35 ± 1.66	0.146
PWTS (mm)	15.90 ± 1.37	16.00 ± 1.66	0.842
LV mass (g)	172.30 ± 39.23	169.76 ± 42.26	0.851
LV index (g/m^2^)	109.85 ± 22.39	112.94 ± 27.46	0.708
LVOT (mm)	21.35 ± 1.42	20.00 ± 2.18	0.030^*^
AOD (mm)	32.35 ± 2.32	30.94 ± 3.27	0.136
LAD (mm)	42.50 ± 4.62	43.94 ± 7.14	0.464
LAV (mL)	50.45 ± 12.10	67.18 ± 28.95	0.024^*^
LAV index (mL/m^2^)	32.80 ± 6.82	45.06 ± 20.20	0.015^*^
E (cm/sec)	48.1 ± 11.7	58.8 ± 17.0	0.031^*^
A (cm/s)	87.9 ± 18.3	82.8 ± 9.8	0.308
DT (ms)	217.70 ± 51.07	207.53 ± 47.41	0.537
PHT (ms)	64.60 ± 15.3	61.82 ± 14.32	0.574
E’-septal (cm/s)	4.70 ± 0.87	5.53 ± 1.50	0.044^*^
E’-lateral (cm/s)	6.15 ± 1.38	7.44 ± 1.31	0.008^**^
E/E’	8.85 ± 2.11	9.24 ± 2.43	0.609
E/A	0.55 ± 0.09	0.67 ± 0.17	0.006^**^
Physical activity (metabolic equivalent-min/week)	2557 ± 1593	1622 ± 766	0.034^*^

Data are expressed as means ± standard deviations. ^*^p < 0.05, ^**^ *p*< 0.01, as tested using the independent *t*-test. LVEF, left ventricular ejection fraction; LVED, left ventricular end-diastole diameter; LVES, left ventricular end-systole diameter; IVSD, interventricular septum at end-diastole; IVSS, interventricular septum at end-systole; PWTD, posterior wall thickness in diastole; PWTS, posterior wall thickness in systole; LVOT, left ventricular outflow tract; AOD, aortic dimension at end-diastole; LAD, left arterial dimension at end-systole; E, early peak mitral inflow velocity; A, late peak mitral inflow velocity; DT, decelerating time; PHT, pressure half time

In contrast, mean values of LVEF, LV end-diastolic diameter, interventricular septum thickness at end-diastole and end-systole, posterior wall thickness in diastole and systole, LV mass, LV mass index, aortic dimension at end-diastole, left arterial dimension at end-systole, A, DT, pressure half time, and E/Eʹ ratio were not significantly different between the two groups.

## 4 Discussion

In this study, the mean LVES was significantly smaller in the metabolic syndrome group than in the normal group. Bhat et al. [17] reported that the LVES, measured using echocardiography, is effective for predicting the recovery of patients with poor LV function. Thus, the smaller the LVES, the more normal the contractile force of the LV. In the present study, metabolic syndrome was determined to not adversely affect the LVES because its value was normal in the normal group and smaller in the metabolic syndrome group. These results are consistent with those of the study by Ayalon et al. [18] in which the LV diameter was not statistically different between the metabolic syndrome and normal groups. However, the LVES can be affected by the aging process as well as by physical activity levels; thus, it cannot be concluded that metabolic syndrome does not affect the LVES. In this study, the mean LVOT (pathway of blood flow from the LV to the aorta) diameter was also significantly larger in the metabolic syndrome group. As LVOT stenosis is an important factor for heart failure, the metabolic syndrome group was expected to have a lower mean value than the normal group [19]. However, both groups showed a normal range of outcome. These results are attributed to the fact that the physical activity level is higher in the metabolic syndrome group. Also, LVOT can be an important factor for functional atrophy in the LV and should be managed in the process of aging; therefore, a future study examining additional effects of physical activity is needed. The present study was limited to determining the effects of physical activity levels on LVOT. The LAV and LAV index were significantly smaller in the metabolic syndrome group than in the normal group. The greater the LAD, the greater the risk of heart failure due to large LAV in patients with atrial fibrillation; previous studies have also reported the association of metabolic syndrome with the LAD [20]. Specifically, previous studies have reported that the LAV index is higher in patients with metabolic syndrome than in healthy individuals [21]. However, the present study showed contradictory results. The results of the present study are limited in explaining that the LV diastolic function is better in the metabolic syndrome group. However, we judged that this result was influenced by physical activity levels in this group.

In this study, the mean E, Eʹ, and E/A ratio values were significantly lower in the metabolic syndrome group compared to the normal group. These values, which reflect the LV relaxation function, were not elevated, despite being exposed to metabolic syndrome. Thus, these values do not reflect the increases associated with changes in cardiac output [22, 23]. Thus, although physical activity levels could not normalize the LV diastolic function, it can be suggested that the levels also did not worsen the function. However, additional research is required because of the limited number of included patients and the short duration of this study.

In this study, the mean physical activity level was significantly higher in the metabolic syndrome group than in the normal group. As a result, the LVOT, LAV, and E values for patients in the metabolic syndrome group, exhibiting high physical activity levels, were significantly better than those for patients in the normal group. However, it can be concluded that higher physical activity levels can restore normal LV function, indicating that physical activity levels in patients may have an indirect effect on the LV function. An additional analysis of the reasons behind the high physical activity levels and specific physical activity, in patients in the metabolic syndrome group is necessary.

This study has some limitations. First, the sample size was low. A future study should recruit more patients to compare physical activity levels among elderly women with and without metabolic syndrome and factors related to LV diastolic function. Second, this study observed only factors related to LV diastolic function. Further study should be conducted to examine heart functions such as cardiac failure and cardiac insufficiency. Third, as patients were recruited from only one health promotion center in Korea, they cannot represent the entire Korean elderly female population. Fourth, the results of this study are limited in explaining the relationship between the LV function and physical activity levels, and further analysis of the training effect on physical activity levels may provide more specific information on the effects of physical activity on LV function. Nevertheless, this study has a strength in that it focused on the LV diastolic function, which has been rarely reported in the field of medicine and public health.

## 5 Conclusion

This study showed that the LV function in elderly women with metabolic syndrome may differ according to their physical activity levels. Women with metabolic syndrome demonstrated higher physical activity levels than normal women. Thus, this higher physical activity level appears to have positively affected the LVES, LVOT, LAV index, E, Eʹ, and E/A ratio values.
